# Gatherings from Grave-Yards; Particularly Those of London; with a Concise History of the Modes of Interment among Different Nations, from the Earliest Periods. And a Detail of Dangerous and Fatal Results Produced by the Unwise and Revolting Custom of Inhuming the Dead in the Midst of the Living

**Published:** 1840-07

**Authors:** 


### ART. X.

*Gatherings from Grave-yards; particularly those of London; with a Concise History of the Modes of Interment among Different Nations, from the earliest periods. And a Detail of Dangerous and Fatal Results produced by the unwise and revolting Custom of Inhuming the Dead in the midst of the Living.* By G. A. WALKER, Surgeon. —*London*, 1839. 8vo, pp. 258.

MR. WALKER's object in the book bearing the above title is to call attention to the present method of disposing of dead bodies, and the many dangers arising therefrom. Custom, in too many instances, reconciles us to bad habits, and even renders it painful to us to separate from them. We are nationally as little disposed to admit, or at least as little disposed to act upon the admission of the objections which apply to burial-grounds in the midst of houses for the living, or to adopt means for the removal of them, as an Irishman is to remove his favorite dunghill, from beneath his family's nose, or to discard from the social circle the domesticated pigs, whose stinks, excrementitious and otherwise, perform so important a part in the early education of at least one of the organs of sense of his family.

Mr. Walker is desirous to render the evils of the present mode of sepulture apparent, being convinced of their enormity, and proportionally anxious for their removal. He is quite an enthusiast in his subject. He says, "Burial-places in the neighbourhood of the living are, in my opinion, a national evil; the harbingers if not the originators of pestilence; the cause, direct or indirect, of inhumanity, immorality, and irreligion." It does not comport with our present purpose to enquire whether Mr. Walker has proved the whole of the above proposition. For such evidence as he has collected, and far and wide has he gone in search of it, we refer to the book itself. Our object will be to select from his volume such matter as belongs to the physical rather than to the moral man.

The author has related many facts, illustrative of the disposition existing in ages long past to bury the dead at a distance from the living. In the first centuries of Christianity, the interment of bodies in towns or churches does not appear to have been practised. Exceptions appear to have been first made about the time of Constantine. Ecclesiastical authorities opposed the practice on the ground of its dangers, and there have been more modern protestations against it.

The French, as is usual in such matters, are far before ourselves in their efforts to obviate the evils arising from the proximity of the dead to the living. And we shall do well to well reflect upon their doings:

"The laws prohibiting interment within cities or towns throughout France were founded upon the reasons urged by the parliament of Paris, viz., that complaints were made of the infectious consequences of the parish cemeteries, especially when the heat of summer had increased the exhalations; that the air was then corrupted; that the most necessary aliments would only keep a few hours in the neighbouring houses; that this proceeded either from the soil being so completely saturated that it could not retain or absorb any longer the putrescent dissolution, or from the too-circumscribed extent of the ground for the number of dead annually interred; and that the same spot was repeatedly used, and, by the carelessness of those who interred the dead, often, perhaps, reopened too soon." (p. 228.)

It is difficult, nay impossible, to estimate the degree in which the population of any place suffers from proximity to dead bodies. We entertain little doubt of the injurious effect of this proximity; but, in the majority of instances, the effect can, of course, be only shown by certain and probably trifling deviations from health. Mr. Walker has accumulated evidence of various kinds; some of this proves the sudden effect of exposure to the atmosphere of vaults and graves. The following is an example: A man was buried in a common grave of the church of Notre Dame, at Montpelier. The grave-digger had scarcely descended into the grave, when he was convulsed, and fell down motionless. A man who followed to draw out the digger, became almost immediately insensible, and was drawn up half dead; it was long before he recovered under the use of strong cordials. Several others were similarly affected by exposure to the same atmosphere. Facts are on record which show the influence of air, infected by the exhalations from dead bodies, in producing fevers of a malignant character. Mr. Walker has mentioned some of this kind; such, for instance, as the following:

"The body of a very fat man was buried in the church of St. Saturnin; twenty-three days afterwards, a grave was opened by the side of the former, to bury a woman there, who had died of the same disease (a mild catarrhal fever). A very fœtid odour immediately filled the church, and affected all those who entered. In letting down the body, the rope slipped, by which the body was shaken; a discharge of sanies followed, the odour of which greatly annoyed the assistants. Of one hundred and seventy persons who entered the church, from the opening of the grave until the interment, one hundred and forty-nine were attacked with a malignant putrid fever, which had some resemblance to the reigning catarrhal fever; but the nature and intensity of the symptoms left no doubt that the malignity was owing *to the infection of the cathedral.*" (p. 97.)

The evidence in cases of this kind is striking and conclusive; but, in some of the instances mentioned by Mr. Walker, he does not make due allowance for causes cooperating with the noxious exhalations.

We are not sufficiently alive to the ill effects which may arise from the interment of bodies in churches, although there can be very few who have not noticed the effect produced upon the atmosphere of a church by the existence of dead bodies beneath.

Haller states, that "a church was infected by the exhalations of a single body twelve years after burial; and that this corpse occasioned a very dangerous disease in a whole convent."

Mr. Walker is disposed to regard the burial of bodies in churches as a frequent cause of epidemic diseases.

It is stated by M. Fourcroy, who was deputed by the French government to superintend the health of workmen employed in certain extensive exhumations, that "the grave-diggers informed him that the putrid process disengages elastic fluid, which inflates the abdomen, and at length bursts it; that this event instantly causes vertigo, faintness, and nausea, in such persons as are unfortunately within a certain distance of the spot where it happens; when the object of its action is nearer, asphyxia and death frequently occur. These men regard this period with the utmost terror; no inducement afforded by the philosophers employed in this superintendence could prevail upon them to assist their researches into this dangerous vapour." (p. 125.)

In addition to the general facts collected by the author, and to which we have very briefly alluded, (for, in truth, the question, as it affects health, lies in a very narrow compass, as, if it can be shown that exhalations from dead bodies have been injurious to the living, a sufficient case is made out for their separation,) Mr. Walker enters into an account of a great number of the metropolitan burial-grounds, and mentions facts connected with their management which call loudly for proper authoritative interference. He has, indeed, undertaken a toilsome and not a very gratifying task. The information contained in his work ought to be extensively known, and especially among those who possess the influence essential to effect the changes which it is his chief object to bring about. Mr. Walker's own residence is in Drury-lane; and he states it as his conviction that the numerous cases of typhus which he there witnesses are mainly to be attributed to the proximity of public as well as of private burial-places. His book must be almost as alarming as the figure of Death himself to the inhabitants of the various districts whose burial-grounds he has examined and described; and if they cannot remedy and are unacquainted with the evil, we feel quite disposed to recommend them to remain in ignorance, as the greater bliss. The connexion of aggravated forms of disease with the exhalations from bodies, is shown to exist on probable not certain evidence. The following is one among the facts which Mr. Walker mentions:

"I was called upon to attend a poor man residing at 33, Clement's-lane; his health was broken, his spirits depressed, and he was fast merging into that low form of fever of which this locality has furnished so many examples. I found him in the back room of an extremely dirty house, his wife and family with him. On looking into the 'green ground,' through the window of his room, I noticed a grave open within a few feet of the house; the sick man replied to my observations: 'Ah, that grave is just made for a poor fellow who died in this house, in the room above me; *he* died of typhus fever, from which his wife has just recovered; *they have kept him twelve days*, and now they are going to put him under my nose, by way of warning to me.' " (p. 152.)

As an instance of the condition of some of the vaults in London churches, that in St. Clement's church is mentioned. The descent to this vault is near the communion table, "and, when opened, the products of the decomposition of animal matter are so powerful, that lighted candles passed through the opening into the vault are instantly extinguished," &c.

Some of these vaults are beneath the churches; others are beneath school-rooms for children; and it may well be questioned whether the modicum of knowledge which they there acquire be not dearly paid for by the effects of such a neighbourhood. It is a curious fact that, although some burial-places have been used as such from time immemorial, they do not appear to change their level. Speaking of the "Green Ground," in Portugal-street, Mr. Walker observes:

"Enormous numbers of dead have been deposited here, and yet the ground remains persistently level. It would be desirable to obtain an account of the number. . . . . . Should it appear that the ground has been overcharged, information should be given of the removal of the earth—necessarily removed to admit of the continuous deposits. Bulk must occupy space. If removals of previous occupants should occasionally have been effected, it would be desirable to know by what authority. . . . . The general question might be asked,

Where have the remains of those removed been deposited—in what manner have the materials of which the coffins were made been used or appropriated? Unfortunately, it has long been notorious, that, through the criminal neglect of survivors, the most disgusting violations of the dead have commonly passed off unnoticed and unpunished. On the surface of many grounds, I have repeatedly seen coffin-wood piled up for removal, and I could not refrain from wondering that this barefaced insult to the living, necessarily preceded by a disgusting exposure and too-frequently dismemberment of the dead, could have taken place with impunity, and that the perpetration of these acts should escape unpunished." (p. 233.)

We have quoted these remarks, because Mr. Walker states that they apply to the "vast majority" of places which he has examined. And yet scenes of this sort, which must be matters of daily observation, occur among a people whose delicate sensibilities are extraordinarily shocked at the idea of a body being employed for the purpose of dissection.

We must now, however unwillingly, terminate our notice of Mr. Walker's book. He has evidently all the enthusiasm of Old Mortality among grave-yards, with a genuine practical benevolent purpose in view; and we take leave of him with an earnest recommendation to continue his efforts, until he witnesses their effects in a change of the present revolting system, and in its miserable effects; promising at the same time that, in as far as is consistent with our purposes as reviewers, we will give him our most cordial cooperation.

---

